# Differentially expressed genes against *Colletotrichum lindemuthiamum* in a bean genotype carrying the *Co-2* gene revealed by RNA-sequencing analysis

**DOI:** 10.3389/fpls.2022.981517

**Published:** 2022-09-15

**Authors:** Maria Jurado, Ana Campa, Juan Jose Ferreira

**Affiliations:** Plant Genetic Group, Regional Service for Agrofood Research and Development (SERIDA), Villaviciosa, Asturias, Spain

**Keywords:** resistance genes, candidate genes, gene ontology, InDel maker, anthracnose

## Abstract

Anthracnose is responsible for large yield losses in common bean crops. RNA-sequencing was used to investigate the differentially expressed genes (DEGs) in response to race 38 of *Colletotrichum lindemuthianum* in two near-isogenic lines (A25 and A4804) that differ in the presence of a resistance gene located in the cluster Co-2. Their responses were analyzed at different hours after inoculation (0, 24, and 48) and within and between genotypes. In all, 2,850 DEGs were detected, with 2,373 assigned to at least one functional GO term. Enriched GO terms in the resistant genotype were mainly related to functions as a response to stimulus, hormone signaling, cellular component organization, phosphorylation activities, and transcriptional regulation. The region containing the *Co-2* cluster was delimited at the end of chromosome Pv11 (46.65–48.65 Mb) through a comparison with the SNP genotypes, obtained using ‘Genotyping by Sequencing,’ among seven resistant lines harboring the Co-2 gene and the susceptible line A25. The delimited region contained 23 DEGs, including 8 typical R genes, that showed higher expression levels in the resistant genotype and non-changes in the susceptible genotype after inoculation. Six R genes encoding protein kinases and an LRR domain formed a cluster in a core region between 46.98 and 47.04 Mb. The alignment of the raw transcriptome reads in the core region revealed structural changes that were used to design four potential breeder-friendly DNA markers, and it revealed some alignments with the intergenic regions, suggesting the presence of genes in addition to those annotated in the reference genome.

## Introduction

The common bean (*Phaseolus vulgaris* L.) is an edible legume crop worldwide. Bean crops can be affected by many diseases (Schwartz et al., 2005), such as anthracnose, caused by the fungus *Colletotrichum lindemuthianum* (Sacc. and Magnus) Lamb-Scrib. In the presence of the fungus and favorable conditions, the yield losses may be significant, reaching 100%. Typical symptoms are deep and well-delimited lesions on hypocotyls, stems, leaf veins, pods, and seeds that usually have salmon-colored spores. Disease progression is favored by humid environments with moderate temperatures, and the disease can lead to plant death. Conidia germinate on the host surface and form a specialized structure, an appressorium, to penetrate the host. Upon entering, the hyphal thread enlarges and penetrates the cells (O’Connell et al., 1985). This process continues for several hours without killing the cells (biotrophic phase). Then, the fungus switches to the necrotrophic phase by producing secondary hyphae, resulting in cell death, which gives rise to cavities that contain acervuli with conidial masses. The conidia can be easily dispersed by splashing raindrops, and the cycle can repeat several times in a growing season. In addition, the conidia can survive in soil, seeds, or plant debris for several years (Tu, 1988, 1992); consequently, an efficient method of managing bean anthracnosis is the use of resistant bean genotypes.

*C. lindemuthianum* exhibits a high level of pathogenic variability. At least 182 races have been reported worldwide from about 1,590 isolates using a standardized set of 12 differential common bean cultivars (Padder et al., 2017). Resistance to anthracnose in common bean essentially follows the gene-for-gene model (Flor, 1971), in which a specific resistance gene protects against specific isolates or races of the pathogen. On the basis of allelism tests and linkage analyses, many anthracnose resistance genes (named *Co-*) have been reported (e.g., Ferreira et al., 2013; Bisneta and Gonçalves-Vidigal, 2020). These anthracnose resistance genes communally show complete dominance, although a few genes with a complementary mode of action have also been identified (Campa et al., 2014, 2017). Anthracnose-resistant loci have been located in specific genetic regions on the bean chromosomes Pv01, Pv02, Pv03, Pv04, Pv07, Pv08, and Pv11. Moreover, genetic mapping of genes conferring resistance to specific isolates revealed that the *Co*- genes were organized in clusters with very close race-specific resistance genes (e.g., cluster Co-3 on Pv04, cluster Co-5 on Pv07, and cluster Co-2 on Pv11, Ferreira et al., 2013; Campa et al., 2014, 2017).

Plants can detect and trigger resistance reactions through the identification of conserved-microbial elicitors using pattern recognition receptors, which gives rise to patterned-triggered immunity (PTI). Plants also have intracellular receptors that identify specific pathogen-virulence molecules and result in effector-triggered immunity (ETI; Dodds and Rathjen, 2010; Meng and Zhang, 2013; Saijo and Loo, 2020). Plant disease resistance genes (*R*) can detect a pathogen attack and facilitate a counter-attack against them. These genes encode for one or several typical protein domains, such as leucine-rich repeats (LRR), nucleotide-binding sites (NB), Toll/Interleukin-1-receptors, coiled coil (CC), transmembrane domain, protein degradation domain, and protein kinase (Afzal et al., 2008; Gururani et al., 2012). In common bean, the positions of the reported Co-clusters co-locate with clusters of *R* genes encoding proteins with kinase or NB-LRR domains (Meziadi et al., 2016). For example, an important cluster of these *R* genes is located at the end of chromosome Pv11 (Meziadi et al., 2016; Bisneta and Gonçalves-Vidigal, 2020). The gene *Co-2*, previously named *Are* and originally reported in the dry bean genotype Cornell 49-242 (Mastenbroek, 1960; Adam-Blondon et al., 1994), has been mapped to this position. Then, using the RIL population Xana/Cornell 49,242, a cluster of specific resistance genes to *C. lindemuthianum* races was mapped to the genetic position of gene *Co-2* (Campa et al., 2014). The anthracnose resistance located in this Co-2 cluster has been widely used in common bean breeding. It was introgressed in the navy bean cultivar Sanilac from Cornell 49-242 (Aylesworth et al., 1983), and in the fabada market class using the resistance sources SanilacBc6Are and A252 (Ferreira et al., 2012). The physical positions of the introgressed genomic region carrying the Co-2 cluster derived from SanilacBc6Are were delimited in the chromosome interval 46.72–48.65 Mb from the genotyping of a set of near-isogenic lines (Ferreira et al., 2017). This region contained 162 annotated genes, of which 70 encoded proteins containing NB-LRR, kinase, or Toll/Interleukin-1-receptors -nucleotide-binding site domains (Ferreira et al., 2017). Thus, the identification of candidate gene(s) involved in the resistance response required further analysis.

To determine the gene controlling specific traits in the genome (candidate gene) based on forward genetic analysis requires the study of large segregating populations, as well as large amounts of genotyping and phenotyping. A comparative transcriptomic analysis of the pathogen–host interactions in resistant and susceptible bean genotypes can provide data on the gene networks involved in the responses, including those mapped on the regions delimited by the genetic analysis. RNA-sequencing (RNA-seq) allows for the investigation of changes in complete transcript sets and their quantification for a specific developmental stage or physiological conditions (Wang et al., 2009). RNA-seq analysis identified 3,250 differentially expressed genes (DEGs) in response to anthracnose race 73 in the isogenic line T-9576 [derived from the cross Jaguar (*Co-1*) × Puebla152 (*co-1*)] through the comparison of susceptible and resistant genotypes (Padder et al., 2016). The DEGs included typical *R* genes and numerous transcription factors (TFs), some of them in or near the region containing the *Co-1* locus. A detailed analysis of this region showed a small cluster of four genes encoding CRINKLY4 kinase in the bean genotypes BAT93 and G19833 (*Phvul.001G243500/KTR1*, *Phvul.001G243600/KTR2*, *Phvul.001G243700/KTR3*, *Phvul.001G243800/KFL*), but an additional gene encoding a truncated and chimeric CRINKLY4 kinase (KTR2/3) was located within this CRINKLY4 kinase cluster in the resistant genotype JaloEEP558 (Cox; Richard et al., 2021). Expression analysis revealed that KTR2/3 is 3-fold up-regulated in JaloEEP558 (Cox) after *C. lindemuthianum* infection compared with the mock control at 24 h post-inoculation, whereas the expression levels of KTR2, KTR3, and KFL were not modified after infection Interestingly, the candidate genes *Phvul.001G243500* and *Phvul.001G243700* were also differentially expressed in response to race 73 in NILs T-9576 (Padder et al., 2016).

In this study, RNA-seq was used to investigate DEGs in response to race 38 of *C. lindemuthianum* with a particular focus on the delimited genomic regions in which the Co-2 cluster is located. The analyses provide data for the gene networks involved in the response to *C. lindemuthianum*, an approach to identifying candidate gene(s) against race 38 in the Co-2 cluster, and the development of markers to accelerate breeding programs.

## Materials and methods

### Plant material

The lines A25 and A4804 were used for the transcriptomic analysis. Line A25 is a selection of the market class fabada (white and very large seeds) that is susceptible to *C. lindemuthianum* race 38 (isolate Cl18). The NIL A4804 is a resistant genotype to *C. lindemuthianum* race 38 obtained from the cross A2806 × X4562. The NILs A2806 and X4562 are derived from A25 (Supplementary Figure 1), both having the seed phenotype of the market class fabada (Ferreira et al., 2012) and resistance to *C. lindemuthianum* race 38 controlled by a gene located in the Co-2 cluster (Ferreira et al., 2017). The resistance gene *Co-2* is derived from Sanilac Bc6Are (Supplementary Figure 1), which was obtained from Cornell 49-242. Finally, sources of the *Co-2* genes, Cornell 49-242 and Sanilac Bc6Are, were also included in this work.

### Inoculation with *Colletotrichum lindemuthianum* race 38

The Cl18 monosporic isolate of *C. lindemuthianum*, classified as race 38 (Ferreira et al., 2008), was used in this work. To obtain abundant sporulation, the isolates were grown at 22°C in darkness for 10 days in potato-dextrose agar (Becton Dickinson and Company). Spore suspensions were prepared by flooding the plates with 5 ml of 0.01% Tween 20 in sterile distilled water and scraping the surface of the culture with a spatula. Inoculations were performed by spraying 10-d-old seedlings with a spore suspension containing 2 × 10^6^ spores mL^−1^. Before sowing, seeds were disinfected in four steps: rinsed in distilled water to remove dirt particles, 30 s in 95% EtOH, 30 s in 15% hydrogen peroxide, and rinsed thoroughly in distilled water. The seedlings were maintained in a climate chamber at 23 to 24°C with 90 to 95% humidity and a 12-h photoperiod.

The experimental design had three replicates (corresponding to three resistance tests), two genotypes (susceptible genotype A25 and resistant genotype A4804), and three treatment assessment times: just before inoculation (named as 0), 24, and 48 h post-inoculation (hpi). On agar media at 24°C, the conidia germinated 4–6 h after sowing, and soon after, the appressoria were observed. At 48 h after sowing, there was extensive hyphal growth on the Petri plates, and a week later, sporulation was observed. Thus, the fungal attack started before 24 hpi, and the plant cells could detect the pathogen and start the cascade of reactions. In susceptible genotypes, after less than 24 hpi, the cytoplasm of infected cells gradually degenerates (O’Connell et al., 1985). Two seedlings per genotype were included in each replicate and treatment. The leaf tissues were harvested, flash-frozen in liquid nitrogen, and stored at −80°C before RNA extraction. In all, the study included 18 samples, named S (susceptible) or R (resistant), follow by the time when the leaf was collected (0, 24, or 48) and then the replicate number (1, 2 or 3). For example, S0.1 represents the susceptible genotype at 0 h/control from experiment 1.

### Total RNA isolation, cDNA library construction, and sequencing

Total RNA was isolated from samples using DNeasy Plant Mini Kit following the manufacturer’s instructions (Qiagen, Germany). RNA was quantified by fluorometric methods and quantity was investigated by using 2,100 Bioanalyzer Instrument (Agilent Technologies, United Kingdom). RNA libraries were prepared using the TruSeq Stranded mRNA Sample Preparation Kit (Illumina) and sequencing was carried out on the Illumina platform. The reads were mapped to the reference genome with HISAT2 splice-aware aligner (Kim et al., 2015) using the bean genome G19833 v1.0 (Schmutz et al., 2014).^1^ Expression profiles are represented as read count and normalization values which were calculated based on transcript length and depth of coverage. The counts for mapped reads were normalized by calculating the FPKM (Fragments Per Kilobase of transcript per Million mapped reads). This analysis was performed in Macrogen Inc. (Seoul, Republic of Korea).

### Differentially expressed genes

A principal component analysis (PCA) and hierarchical clustering analysis (HCA) were performed to detect the possible sources of noise in the results. The DEGs were identified through comparisons within resistant (A4804) and susceptible genotypes (A25) at 0 and 24 hpi and at 0 and 48 hpi (comparisons named as R24-R0, R48-R0, S24-S0, and S48-S0). In addition, the DEGs were investigated through comparisons between the two genotypes at 0, 24, and 48 hpi (named R0-S0, R24-S24, and R48-S48). The NOISeq package (2.38.0; Tarazona et al., 2011) and pheatmap 1.0.12 package in R project (R Core Team, 2021) were used to explore the quality of the samples and detect DEGs. The DEGs were identified using the criterion q > 0.80. Specific and common DEGs between genotypes and treatments were visualized using Venn diagrams constructed using the package ggVenn/ggplot2 in R project.

### Gene ontology (GO) analysis of DEGs

To investigate functional groups of DEGs in response to fungus, a GO analysis was performed using the Ensembl database (organism dataset: pvulgaris_eg_gene, version 2022-02-10) considering the three categories: biological process (BP), molecular function (MF), and cellular components (CC). The GO enrichment of significantly over-represented terms was carried out using the R package ViSEAGO (Brionne et al., 2019) and the DEG list. Enrichment tests were assessed using Fisher’s exact test (*p* ≤ 0.01) for the resistant and susceptible genotypes at both time level datasets [24 hpi (S0-S24, R0-R24) and 48 hpi (S0-S48 R0-R48)] and the three GO categories. The enriched GO terms were grouped on the basis of Wang’s semantic similarity into functional clusters using hierarchical clustering between GO terms with GO graph topology and Ward’s criterion.

### Genotyping by sequencing

The bean lines Cornell 49-242, Sanilac Bc6Are, A25, A2806, X4562, and A4804 were genotyped using the Genotyping by Sequencing method (Elshire et al., 2011) optimized in accordance with Schröder et al. (2016). DNAs were isolated from young leaves following the SILEX method (Vilanova et al., 2020), and DNA quality was checked in agarose gels. Genomic DNAs from the lines were digested individually with the *Taqα1* and *MseI* restriction enzymes. Libraries were built based on the Poland et al. (2012) protocol with a different adaptor for ligation. Then, individual samples were checked by PCR, and the resulting products were visualized on 2% agarose gels. In total, 20 barcoded samples were pooled for PCR amplification. Sequencing was performed in the Illumina platform by Macrogen Inc. (Seoul, Republic of Korea). SNP calling was carried out by AllGenetics&Biology SL^2^ using the *P. vulgaris* reference genome (genotypes G19833 v1.0,^3^ Schmutz et al., 2014). The SNPs supplied by GBS were filtered and extracted using TASSEL 5.1 software (Bradbury et al., 2007). SNPs with the following characteristics were considered in the analysis: (i) missing data, less than 50%; (ii) minor allele frequency, greater than 5%; (iii) mapped to one of the 11 pseudo-chromosomes. The genomic regions were delimited based on the physical position of the SNPs flanking the introgressed region (coordinates of the first and last introgressed SNPs). Finally, the GBS results reported by Murube et al. (2017) for the NILs A1258, A2806, and X2776 were used.

### RNA-seq reads assembly and visualization

To identify variations useful to develop specific markers for the Co-2 region, raw RNA-seq reads of each experiment were assembled with the chromosome Pv11 of *P. vulgaris* v1 using the RBowtie package (Langmead et al., 2009). The alignments were visualized in Integrative Genomics Viewer software (Robinson et al., 2011), and the selected region was explored to detect polymorphism, insertions, or deletions (InDel), between the reference genome and the studied genotypes. The observed polymorphisms were also assessed by BLASTN and ClustalW alignments with the five bean genomes available (*Phaseolus vulgaris* G19833 v2.1; *Phaseolus vulgaris* 5 -593 v1.1; *Phaseolus vulgaris* UI111 v1.1; *Phaseolus vulgaris* Labor Ovalle v1.1).^4^ Those regions that showed an InDel in one genome were considered.

The primers for the PCR amplification of regions containing InDels were designed using the PrimerBlast tool (Ye et al., 2012). PCR reactions (20 μl) contained 2 μl of PCR buffer (10×), 1.2 μl MgCl2 (25 mm), 2 μl dNTP mixture, 0.8 μl of each primer (10 μm), 8.05 μl distilled water, 0.15 μl of TaKaRa LA Taq DNA Polymerase (5 U/μl; TaKaRa), and 50 μl 10 ng/μl DNA. The PCR reaction protocol performed on a Verity Thermal Cycler (Life Technologies Carlsbard, CA, United States) was as follows: an initial 5 min at 95°C; 30 cycles of 30 s at 95°C, 45 se at 62°C, 60 s at 72°C; final extension step at 72°C for 10 min. PCR products were separated on a 2% agarose gel, stained with RedSafe^™^ Nucleic Acid Staining Solution (iNtRON, Seoul, Republic of Korea), and visualized under ultraviolet light.

## Results

### Transcriptome sequencing of resistant and susceptible genotypes

**Figure 1:**
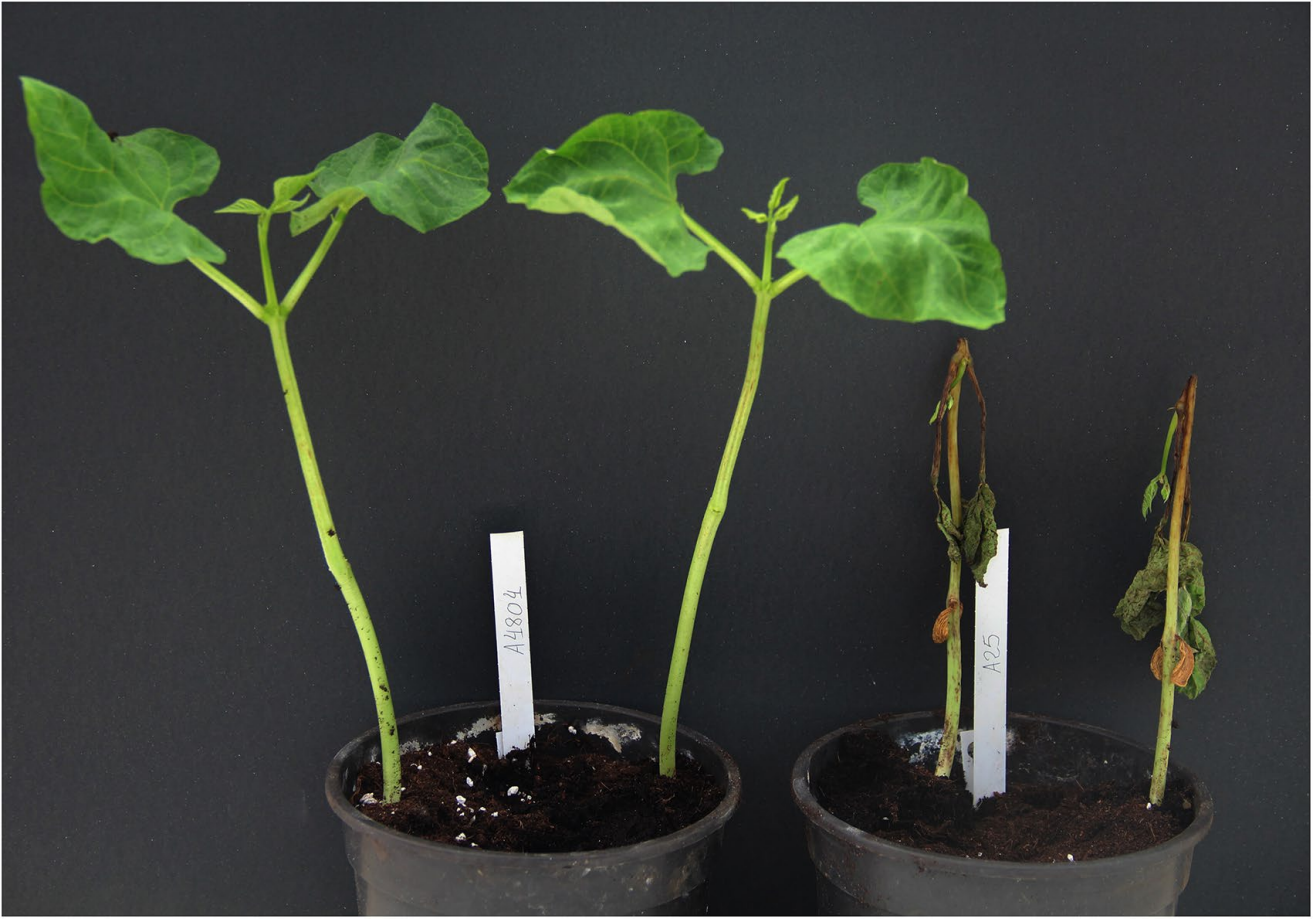
Reactions of the genotype A4804 (left; resistant) and A25 (right; susceptible) against monosporic isolates Cl18 (race 38) 7 days after inoculation.

At 7 days post-inoculation with *C. lindemuthianum* race 38, the susceptible genotype (A25) was dead, whereas symptoms were not observed on the resistant genotype A4804 (Figure 1). A total of 18 cDNA libraries were generated, one per genotype, replicate, and treatment, and, in total, they generated 876,756,658 clean reads using the NovaSeq platform (Supplementary Table 1). The reads of all the samples were used for transcriptome assembly, and an average of 91.1% of reads were mapped to the reference genome. Mapped reads were normalized by calculating the FPKM. A PCA of FPKMs estimated for each genotype, replicate, and treatment revealed two components that explained 79% of the variation. The biplot shows 3 samples, S0.1, S24.1, and R24.1, separated from the remaining 15 samples (Supplementary Figure 2A). Similarly, a hierarchical clustering analysis (HCA) classified the samples into two different main clusters separated from the samples S0.1, S24.1, and R24.1 (Supplementary Figure 2B). These samples had low RNA Integrity Numbers (RIN), less than 5.7, in the RNA extraction and were discarded from the analysis.

### Differentially expressed genes

**Figure 2:**
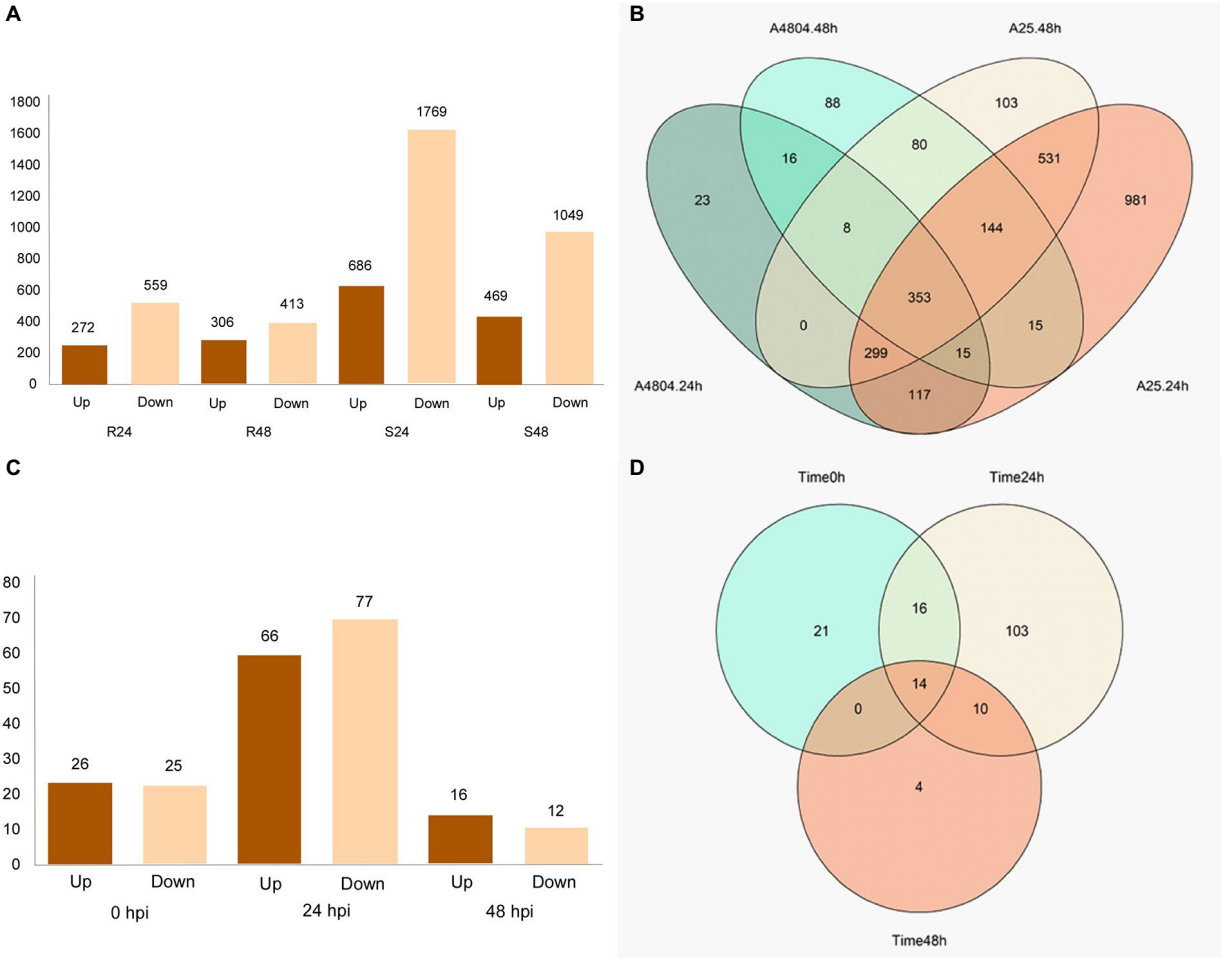
Visualization of DEGs detected from comparison at different hpi and between genotypes in the same hours post-inoculation (hpi). **(A)** Histograms showing the number of DEGs, upregulated and downregulated, at different hpi in the two genotypes **(B)** Venn diagrams showing the numbers of specific and common DEGs at different hpi in the two genotypes. **(C)** Histograms showing the number of DEGs, upregulated and downregulated, at the same hpi. **(D)** Venn diagrams showing the numbers of specific and common DEGs at different hpi.

The DEGs were identified from seven comparisons (R24-R0, R48-R0, S24-S0, S48-S0, S0-R0, S24-R24, and S48-R48). A total of 5,740 differential expressions, involving 2,850 unique genes, were identified (Supplementary Table 2). A higher number of DEGs were found in the susceptible genotype than in the resistant genotype (Figure 2A) when the expression levels at 24 and 48 hpi were compared with the control (0 hpi). At 24 hpi after the susceptible genotype A25 was inoculated with *C. lindemuthianum*, 2,455 DEGs (686 upregulated; 1,769 downregulated) were detected, and the number decreased to 1,518 (469 upregulated; 1,049 downregulated) at 48 hpi (Figure 2A). Among the DEGs in the susceptible genotype, 1,615 genes only appeared in this genotype (Supplementary Figure 2B). The resistant genotype A4804 showed 831 DEGs (272 upregulated; 559 downregulated) at 24 hpi and 719 DEGs (306 upregulated; 413 downregulated) at 48 hpi. In addition, 127 DEGs were only detected in the resistant genotype, and 16 of them were identified at both 24 and 48 hpi (Figure 2B).

For the comparisons between resistant and susceptible genotypes at the same hpi, the highest number of DEGs, 143 (66 upregulated; 77 downregulated), were detected at 24 hpi. The number of DEGs decreased to 28 (16 upregulated; 12 downregulated) at 48 hpi (Figure 2C). In total, 21 genes were differentially expressed between both genotypes before inoculation and were discarded from the analysis. There were 14 DEGs between the resistant and susceptible genotypes that were common for the three hpi (Figure 2D). In contrast, there were 168 DEGs in both genotypes after inoculation, 10 of which maintained the differential expression at 24 and 48 hpi (Figure 2D): *PHAVU_003G011800g, PHAVU_004G005400g, PHAVU_004G046400g, PHAVU_004G094000g, PHAVU_007G216700g, PHAVU_008G103500g, PHAVU_010G012900g, PHAVU_011G044000g, PHAVU_011G201700g, and PHAVU_011G203000g*. Finally, four DEGs were only detected between both genotypes at 48 hpi: *PHAVU_001G083000g, PHAVU_003G011000g, PHAVU_003G0939001g,* and *PHAVU_007G276500g.*

### Functional classification of DEGs

A GO analysis was performed using the DEGs in each genotype. Among the 2,850 DEGs, 2,373 were assigned to at least one functional GO term in the Ensembl database (analysed 23/02/2022).^5^ To reveal the functional processes involved in the resistant and susceptible genotypes, the DEGs at 24 and 48 hpi were analyzed for enriched terms in the three GO categories (Supplementary Figures 3A–F, 4A–F). The differences were more evident at 48 than at 24 hpi, particularly in the BP category. The BP category contained 41 enriched GO terms at 24 hpi (Supplementary Table 4), 25 in the resistant genotype and 39 in the susceptible, with ‘translation’ and ‘biosynthetic process’ being the most significant pathways at both times. At 48 hpi, 111 GO terms were enriched (Supplementary Table 3), 89 in the resistant genotype, with ‘response to biotic stimulus,’ ‘cell wall organization,’ ‘hormone signalling,’ ‘flavonoid metabolism,’ ‘dephosphorylation,’ and ‘phosphatase activity’ being highly featured, and 31 in the susceptible genotype, with ‘translation’ and ‘biosynthetic process’ being the more prevalent terms. For MF at 24 hpi (Supplementary Table 4), only nine GO terms were enriched, four in the resistant and eight in the susceptible genotypes. In both genotypes, the term ‘structural molecule activity’ was the most enriched. At 48 hpi, 34 GO terms were significant (Supplementary Table 3) in MF, 31 in the resistant and 14 in the susceptible genotypes. Structural ‘molecule activity’ was the most enriched process in the susceptible genotype, whereas ‘hydrolase’ and ‘signaling receptor activity’ were the most enriched processes in the resistant genotype. For the CC category, 19 GO terms were enriched at 24 hpi (Supplementary Table 4), 13 of them in the resistant genotype and 14 in the susceptible genotype. The GO term ‘organelle ribosome’ was the most enriched in both cases, with ‘non-membrane-bounded organelle’ also being highly enriched in the resistant genotype. At 48 hpi 26 GO terms were enriched (Supplementary Table 3), 14 in the resistant genotype, with ‘extracellular’ and ‘cell wall regions’ being the most enriched term, and 15 in the susceptible, with ‘ribosome’ being the most enriched term.

### The physical position of the Co-2 cluster in the line A4804

Sequencing the GBS libraries yielded approximately 17.7 million reads in the six genotypes (Cornell 49-242, SanilacBc6Are, A25, A2806, X4562, and A2804), resulting in a total of 108,593 SNPs and 35,244 SNPs after filtering. The GBS revealed that 506 SNPs mapped on the end chromosome Pv11 (physical position>45 Mb). Genotypic comparisons of these 506 SNPs among the three resistant NILs harboring the *Co-2* gene (A2806, X4562, and A2804), the resistance sources Cornell 49-242 and Sanilac Bc6Are, and the susceptible line A25 revealed that the three resistant NILs exhibited an introgressed region at the end of chromosome Pv11. These regions are tagged by SNPs having the donor resistance genotype (Sanilac Bc6Are). On the basis of the physical position of these SNPs, line A2806 has a region located at 45.10–48.78 Mb tagged by 183 SNPs of the SanilacBc6Are genotype (except for 21 isolated SNPs). The lines X4562 and A4804 maintained an introgressed region at 46.65–50.32 Mb that was tagged by 132 SNPs of the SanilacBc6Are genotype (Supplementary Figure 4). There was a common introgressed region among the three NILs located between 46.65 and 48.65 Mb.

### DEGs in the genomic Co-2 region

**Figure 3:**
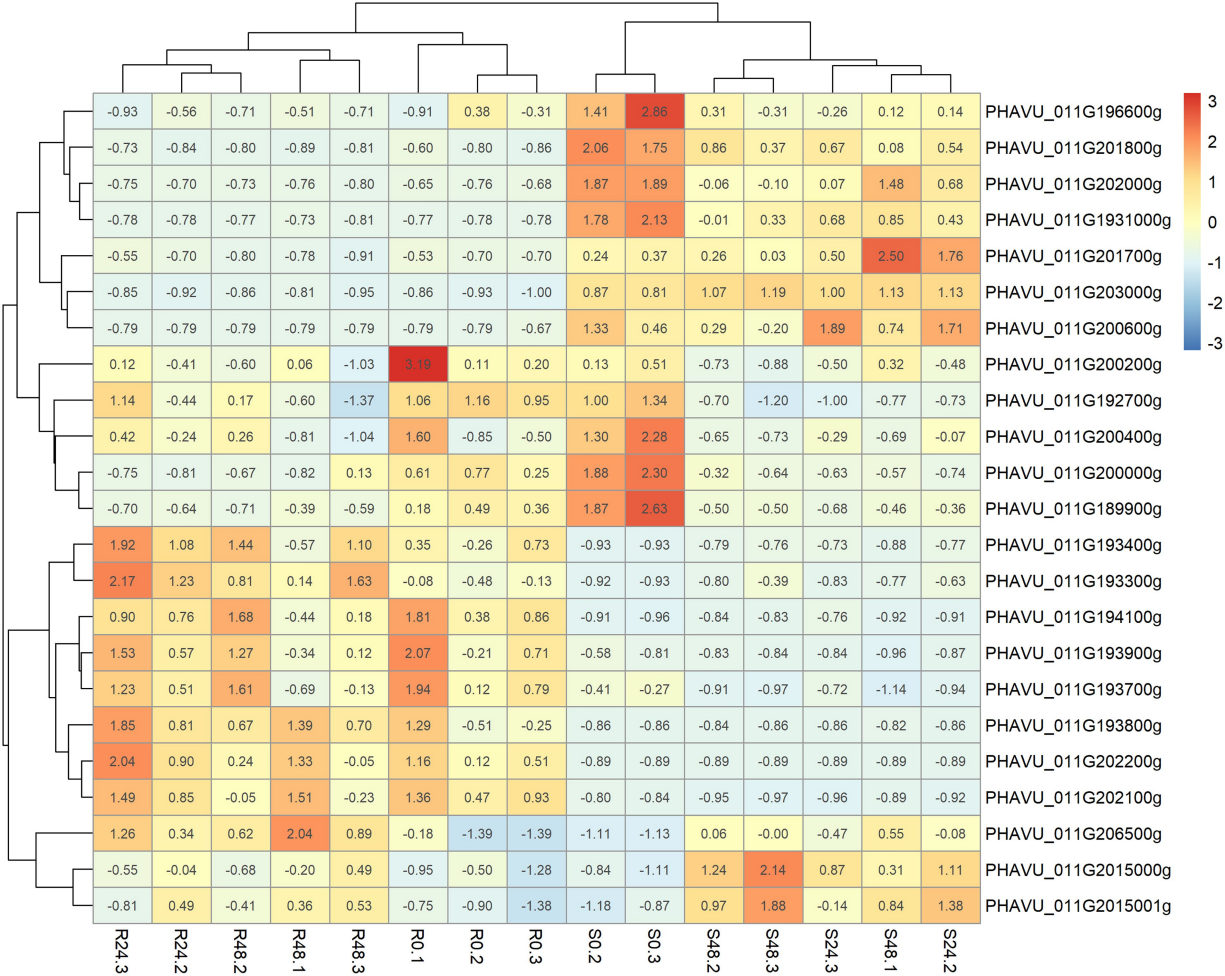
Heatmap built with the package pheatmap showing the expression (FPKM) for the resistant and susceptible lines (A4804 & A25) per hour post-inoculation and sample in the 23 DGEs located in the delimited region introgressed with the resistance to *C lindemuthinanum* race 38 in the genotype A4804.

The introgressed region in line A4804 carrying the *Co-2* gene (46.65–48.65) has 165 annotated genes. Among them, 23 were differentially expressed in response to *C. lindemuthianum*. The representation of the differential expressions of the 23 genes in a heatmap revealed a separation between the resistant and susceptible genotypes (Figure 3) and the following four main groups of genes:

Group I, includes seven annotated genes that showed higher expression levels in the susceptible genotype and no changes in the resistant genotype, in response to *C. lindemuthianum*: *PHAVU_011G196600g, *PHAVU_011G201800g*, *PHAVU_011G201700g*, *PHAVU_011G202000g*, *PHAVU_011G193100g, *PHAVU_011G203000g*, and *PHAVU_011G200600g.* The genes marked with an asterisk encode hypothetical proteins with LRR domains.

Group II, contains five annotated genes that decreased in expression in response to *C. lindemuthianum* in both susceptible and resistant genotypes: *PHAVU_011G200200g*, *PHAVU_011G192700g*, *PHAVU_011G200400g*, *PHAVU_011G200000g*, and *PHAVU_011G189900g.*

**Figure 4:**
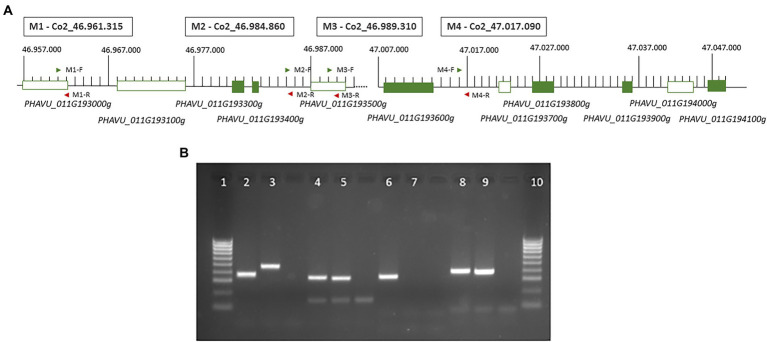
**(A)** Representation of the ‘core region’ with the 6 annotated genes in the reference genome and the position of the four markers developed in this study. Green boxes represent candidate genes revealed in transcriptomic analysis (Figure 3). **(B)** Agarose gel (2%) showing the results of the PCR amplification for the four markers developed: lines 1 and 10, marker 100 bp; 2 and 3, results in n A25 and A4804 for the marker M1_ Co2_46.961.315; 4 and 5, results in A25 and A4804 for the marker M_ Co2_46.984.860; 6 and 7, results in A25 and A4804 for the marker M3_ Co2_46.989.310; 8 and 9, results in A25 and A4804 for the marker M4_ Co2_47.017.090.

Group III, includes eight genes with higher expression in the resistant genotype and non-changes in the susceptible genotype in response to *C. lindemuthianum*: *PHAVU_011G193400g*, *PHAVU_011G193300g*, *PHAVU_011G194100g*, *PHAVU_011G193900g*, *PHAVU_011G193700g*, *PHAVU_011G193800g, *PHAVU_011G202200g*, and *PHAVU_011G202100g. These genes have a serine/threonine-protein kinase function (6) or encode proteins with LRR domains (marked with an asterisk). They are located near two positions: a region with six genes (46.98–47.04 Mb, ‘core region’; Figure 4A) and another region with two genes (48.02 Mb).

Group IV, includes three annotated genes that tend to increase their expression in response to *C. lindemuthianum*, particularly in the susceptible genotype: *PHAVU_011G206500g*, *PHAVU_011G2015000g*, and *PHAVU_011G2015001g.* These genes have unknown functions in the bean reference genome.

### Specific markers for the Co-2 cluster

The assembly of the raw transcriptome reads in the ‘core region’ in the chromosome Pv11 revealed InDels when read sequences of the resistant genotype (A4804) and the reference genome were aligned. The occurrence of InDel was also checked by BLASTN with the four available bean genomes and five of InDels were found in at least one of the genomes available. The susceptible genotype A25 did not show changes with the reference genome (G19833) in these four regions. The four polymorphic positions with InDel were:

M1 position, located in the third exon of the gene *PHAVU_011G193000g* (see Figure 4A). The resistant genotype A4804 has an insertion of 18 bp, also observed in the genomes 5–593 v1.1, UI111 v1.1, and, Labor Ovalle v1.1 (Supplementary Figure 5A).

M2 position, located in the intergenic region between the DEG *PHAVU_011G193400g* (Figure 4A) and *PHAVU_011G193500g*. The genotypes A4804, 5–593 v1.1, and UI111 v1.1 have a deletion of 10 bp. The reads with these sequences aligned with the gene Pv5-593.11G192900 annotated in the bean genome 5–593 (Supplementary Figure 5B).

M3 position, located in the gene *PHAVU_011G193500g*. The genotypes A4804 and 5–593 show a deletion of 6 bp and closed a mutation of two bp (Supplementary Figure 5C).

M4 position, located in the intergenic region between the genes *PHAVU_011G193600g* and *PHAVU_011G193700g* in the reference genome, but is annotated in the version 2 of G19833 in the gene *Phvul.011G193600.1*. The genotypes A4804, 5–593, and UI111 have a 12 bp deletion (Supplementary Figure 5D).

Specific primers were designed to amplify the four regions containing the InDels (Supplementary Table 5). PCR amplification generated fragments of the expected sizes, and the four markers were polymorphic between the resistant and susceptible genotypes A4804 and A25 (Figure 4B). The markers M1, M2, and M4 exhibited variations in size, whereas the M3 marker showed an amplification/non-amplification variation. These polymorphisms were also observed among the resistant genotypes Cornell49242, SanilacBc6Are, A2806, X2776, and X4562 (all of them with genotype as A4804), as well as the susceptible genotype A25.

## Discussion

Anthracnose is an important disease of common bean that causes significant losses worldwide (Mohammed, 2013). Resistance to anthracnose in common bean has been extensively studied through genetic analysis in segregating populations and many resistance genes have been described (Co-genes; Ferreira et al., 2013). However, information on the molecular responses to specific plant–fungus interactions is limited. Comparative transcriptome analyses have been used to study responses to disease in plants and to identify the specific genes involved (Kankanala et al., 2019), but they have not been extensively applied to investigate resistance to bean anthracnose (Oblessuc et al., 2012; Padder et al., 2017). In this study, the changes in the transcriptomic profile during the response to *C. lindemuthianum* race 38 were investigated in two NILs, the susceptible line A25 and the resistant line A4804 carrying a resistance gene located in the Co-2 cluster.

A total of 2,850 DEGs involved in the response to *C. lindemuthianum* were detected in this study. More DEGs were found in the susceptible genotype than in the resistant genotype, which corroborated the results of Padder et al. (2016) in response to race 73. However, the resistant genotype showed a higher number of enriched GO terms than the susceptible genotype (Supplementary Tables 3, 4), indicating the diversity of processes involved in the resistance response. At 48 hpi, the resistant genotype had significantly enriched GO terms for biological functions related to cellular events typically involved in response to stresses, such as cell wall biogenesis (e.g., GO:0044036, GO:0071555, GO:0042546; see Supplementary Figure 3A) and hormone network regulation (GO:0009737, GO:0009738, GO:0071215), as well as transcriptional, translational, and metabolic reprogramming. Notably, there was a great enrichment of phosphate inhibitor and regulation activities (GO:004864, GO:0019212) in the resistant genotype at 48 hpi, which represents a mechanism of response in plants reported in other species (Rakwal et al., 2001; Alvarado-Gutiérrez et al., 2008). In addition, DEGs involved in the regulation of protein phosphorylation and dephosphorylation (e.g., GO:0035305, GO:0016311, GO:0045936, GO:0010921) were identified as mitogen-activated protein kinases (GO:0004672). Protein kinases play crucial roles in plant resistance to pathogens because they are involved in signaling downstream of receptors/sensors that transduce extracellular stimuli into intracellular responses in eukaryotes (Meng and Zhang, 2013).

Plant hormones, such as salicylic acid, jasmonic acid, abscisic acid, and ethylene, also play important roles in plant disease resistance (Mauch-Mani and Mauch, 2005). For instance, cytokinin and ethylene responses were upregulated, whereas jasmonic acid, gibberellin, and abscisic acid responses were downregulated in response to *C. lindemuthianum* (race 73) in the genotype SEL1308 (Oblessuc et al., 2012). The terms GO:0009738 and GO:0009737 (abscisic acid-activated signalling pathway) were significantly enriched in this analysis and involved 14 genes (Supplementary Table 3A). The resistant genotype was enriched for GO terms involving salicylic acid (e.g., GO:2000031, *PHAVU_005G047200g*) and ABA (e.g., GO:0010427, *PHAVU_003G109000g*). Phytohormone networks are connected through crosstalk involving TFs or sequence-specific DNA-binding factor proteins that control the transcription rates of specific genes (Monson et al., 2022). The resistant genotype was enriched for terms, such as GO:0006355 and GO:1903506 (regulation of transcription), which involved a lot of genes. The role of TFs in the response to anthracnose was previously reported by Padder et al. (2016) and verified in this analysis. The following DEGs that code for TFs were identified in the resistant genotype (Supplementary Tables 2, 3A): *PHAVU_002G056300g*, *PHAVU_002G260700g*, and *PHAVU_002G265400g* (Supplementary Table 3; GO:0003700). DEGs *PHAVU_002G056300g* and *PHAVU_002G265400g* were also identified in response to race 73 (Padder et al., 2016).

The genotyping of the NILs A2806, A4804, and X4562 through GBS allowed the introgressed region with the *Co-2* gene at the end of chromosome Pv11 to be delimited. The SNPs tagging this genomic region share the resistance donor’s genotype (Sanilac Bc6 Are). This position co-located with the anthracnose resistance genes, forming the cluster Co-2. The resistance loci to races 6, 38, and 39 were mapped between makers IND11_46.8842 and Pv11_4600a at physical positions 46.8 and 47.07 Mb in the RIL Xana/Cornell (unpublished data). The size and position of this region were similar to those reported by Murube et al. (2017) in the NILs A2806 and X2776. The lines X4562 (derived from X2776) and A4804 (from X2776 x A2806) maintained the introgressed region of the line X2776, between 46.65 and 50.10 Mb (see Supplementary Figure 4). The results showed a common introgressed region of ~2 Mb (46.65–48.65 Mb) among the resistant NILs A2806, A4804, X2776, and X4562. This common region also overlaps with the introgressed region in the NIL A1258 (46.65–48.65 Mb) obtained from a backcrossing program in which the line A252 was the donor parent and the line A25 the recurrent parent. The breeding line A252 has a resistance cluster mapped at the end of the chromosome Pv11 that includes a resistance locus to race 38 (Rodríguez-Suárez et al., 2007). The NIL A1258 has a large introgressed region in chromosome Pv11 (Murube et al., 2017). The resistant NILs A1258, X2776, A2806, X4562, and A4804, all with resistance loci in the Co-2 cluster, have a common region between 46.65 and 47.07 Mb. A differential expression analysis revealed 23 DEGs in this region (Figure 3), including a group of 8 genes (cluster III) with higher expression levels in the resistant genotypes and non-changes in the susceptible genotype after *C. lindemuthianum* inoculation. Interestingly, six of these eight genes form a cluster at 46.98–47.04 Mb, at the border of the common regions among the six NILs (Supplementary Figure 4): *PHAVU_011G193300g*, *PHAVU_011G193400g*, *PHAVU_011G193700g*, *PHAVU_011G193800g*, *PHAVU_011G193900g,* and *PHAVU_011G194100g*. All of these genes are typical *R* genes with a serine/threonine protein kinase or LRR domain that may be associated with the initiation of plant defense response signals and with pathogen recognition (Dodds and Rathjen, 2010; Meng and Zhang, 2013; Monson et al., 2022). The genomic region containing the six DEGs (core region) is the main candidate region for the resistance gene(s) to race 38.

RNA-seq technologies are powerful tools for studying gene expression, but they have limitations when using a unique genome as a reference because the annotated genes can vary between databases and genomes. Exploring RNA-seq data can be useful to improve the annotation of genetic variants (Chen et al., 2017). Da Silva et al. (2013) discovered 1,873 new genes in a local grapevine variety not annotated in the reference genome, and Tisserant et al. (2011) mapped 13% of the cDNA reads outside the predicted UTRs and gene models. Within the *P. vulgaris* species differences in the numbers of annotated protein-coding genes (e.g., 27,433 in G19833; 27,065 in 5–593) can be found; therefore, the appearance of RNA-seq reads in the ‘core region’ aligned to the intergenic regions (e.g., positions M2 and M4) of the reference genome can be treated and explored in later studies as putative novel genes/transcripts. Because genome-guided or *de novo* transcriptome reconstruction is needed to annotate these possible new genes, we only used the polymorphisms found in other *P. vulgaris* genomes to design markers and explore the genetic variation. Position M2 only corresponds with an annotated gene in the bean genome 5–593. This gene encodes a protein kinase (*Pv5-593.11G192900*), and an additional kinase gene in the Co-1 cluster, implicated in the defense against *C. lindemuthianum* race 100 in common bean, has been reported by Richard et al. (2021). Reads aligned in the M4 position revealed the problem of using only one database as a reference, because when the G19833 v1.1 genome in NCBI is used as a reference to record gene expression levels, this region is not a gene. However, it is the gene *Phvul.011G193600.1* in the G19833 v2.1 genome in the Phytozome database. As a result, the possible differences in expression are not explored in this gene, which encodes an LRR protein, included in the ‘core region.’ These alignments did, however, reveal polymorphisms that were used to develop four potential new breeder-friendly DNA markers. The most popular markers linked to the *Co-2* gene are SCH20 and SCAreoli (Geffroy et al., 1998), which are both Cleaved Amplified Polymorphic Sequences in which the polymorphism was revealed after a restriction enzyme cut. The polymorphisms in the four developed markers can be visualized in agarose gels, with the M1 marker showing size variations between the resistant and susceptible genotypes used in this work.

## Conclusion

This study shows that by combining the physical locations and a comparative transcriptome analysis, a closer approximation of the region containing the candidate genes controlling resistance was possible. This approach allowed us to reduce an initial delimitated region of ~2 Mb to a ‘core region’ of ~60,000 bp. The ‘core region’ contains nine annotated genes in the G19833 genome, six of which were differentially expressed in response to *C. lindemuthianum.* They encode protein kinase or LRR domains, typical of *R* genes. However, additional resistance genes can also be present in the ‘core region’ of the resistant genotype as revealed by the alignments of obtained reads in intergenic regions of the bean reference genome. In addition, that alignments showed a major InDel that was used to design functional markers to help accelerate breeding programs and genetic analyses.

## Data availability statement

The original contributions presented in the study are publicly available. This data can be found at: https://www.ncbi.nlm.nih.gov/bioproject/ PRJNA851559.

## Author contributions

MJ performed the transcriptomic and bioinformatic analysis. AC performed the resistance test and part of the transcriptomic analysis. JJF conceived the work and write the manuscript. All authors contributed to the article and approved the submitted version.

## Funding

This project has received funding from the European Union’s Horizon 2020 research and innovation program under grant agreement No 774244 (BRESOV). MJ is supported by the Grant PRE2019-091249 funded by MCIN/AEI/10.13039/501100011033 and, as appropriate, by “ESF Investing in your future” or by “European Union NextGenerationEU/PRTR.”

## Conflict of interest

The authors declare that the research was conducted in the absence of any commercial or financial relationships that could be construed as a potential conflict of interest.

## Publisher’s note

All claims expressed in this article are solely those of the authors and do not necessarily represent those of their affiliated organizations, or those of the publisher, the editors and the reviewers. Any product that may be evaluated in this article, or claim that may be made by its manufacturer, is not guaranteed or endorsed by the publisher.

## Footnotes

^1^ https://www.ncbi.nlm.nih.gov/genome/380

^2^ www.allgenetics.eu

^3^ https://www.ncbi.nlm.nih.gov/genome/380

^4^ https://phytozome-next.jgi.doe.gov/

^5^ www.ensembl.org
