# Supplementary material for: Differentially expressed genes against Colletotrichum lindemuthiamum in a bean genotype carrying the Co-2 gene revealed by RNA-sequencing analysis
Source: Front Plant Sci. 2022 Sep 15;13:981517. doi: 10.3389/fpls.2022.981517 (PMC9615912; doi:10.3389/fpls.2022.981517)
Supplement: Supplementary file 4 [file Data_Sheet_1.pdf]

Supplementary Material

Differentially expressed genes against *Colletotrichum lindemuthiamum* in a bean genotype carrying the *Co-2* gene revealed by RNA-sequencing analysis

Maria Jurado, Plant Genetic Group, Regional Service for Agrofood Research and Development (SERIDA), 33300, Villaviciosa, Asturias, Spain.

https://orcid.org/0000-0002-9776-7296

Ana Campa, Plant Genetic Group, Regional Service for Agrofood Research and Development (SERIDA), 33300, Villaviciosa, Asturias, Spain.

https://orcid.org/0000-0003-3970-9079

Juan Jose Ferreira, Plant Genetic Group, Regional Service for Agrofood Research and Development (SERIDA), 33300, Villaviciosa, Asturias, Spain. jjferreira@serida.org

https://orcid.org/0000-0002-8782-8868

For correspondence: jjferreira@serida.org

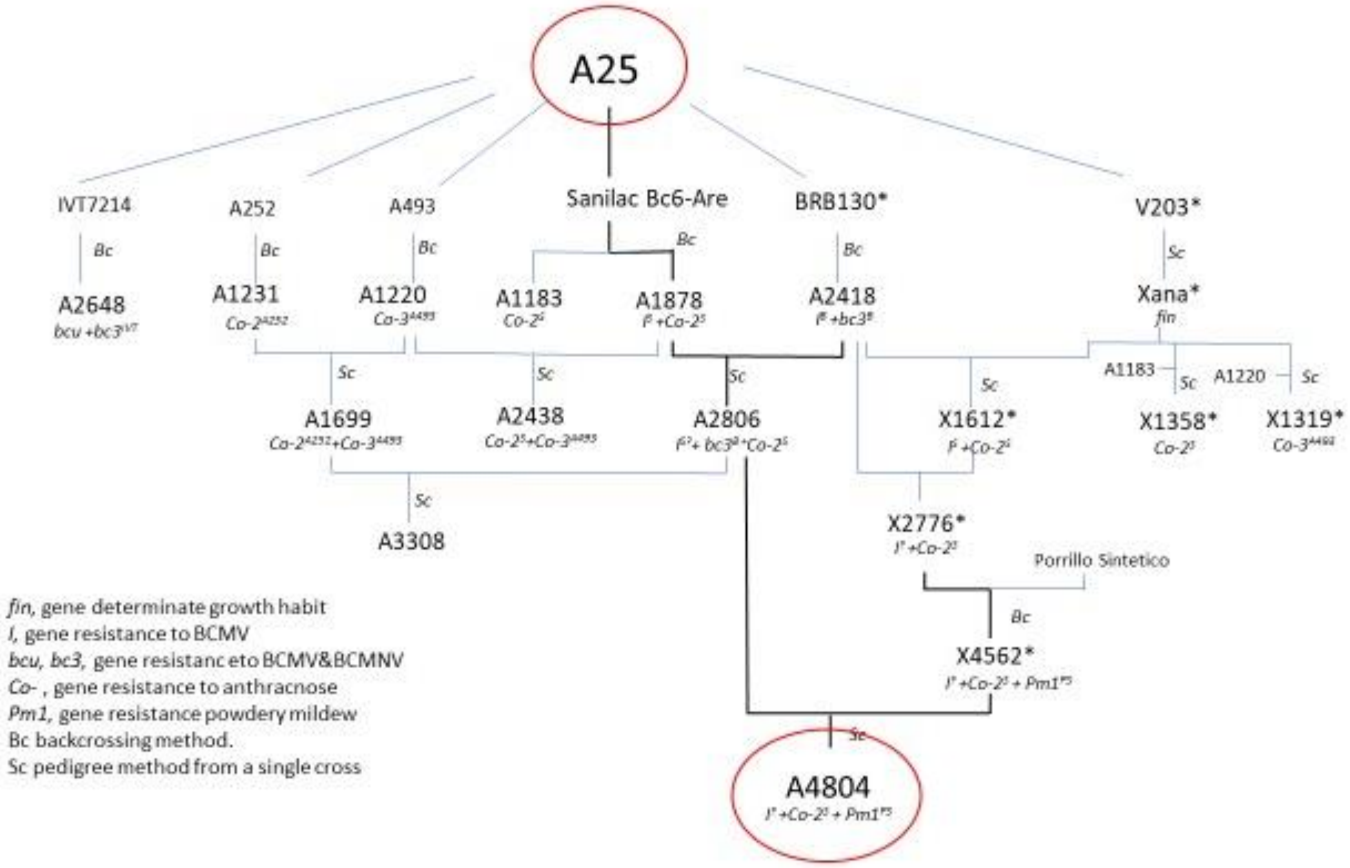

Supplementary Figure 1. Pedigree of lines analyzed in this work. Line A25 proceeds from a selection of the landrace Andecha, classified in the market class fabada. Line A4804 is an isogenic line originally derived from A25 carrying a resistance locus to anthracnose located in the cluster Co-2. Additionally, line A4804 carries resistance to BCMV (gene I) and powdery mildew (gene Pm1)

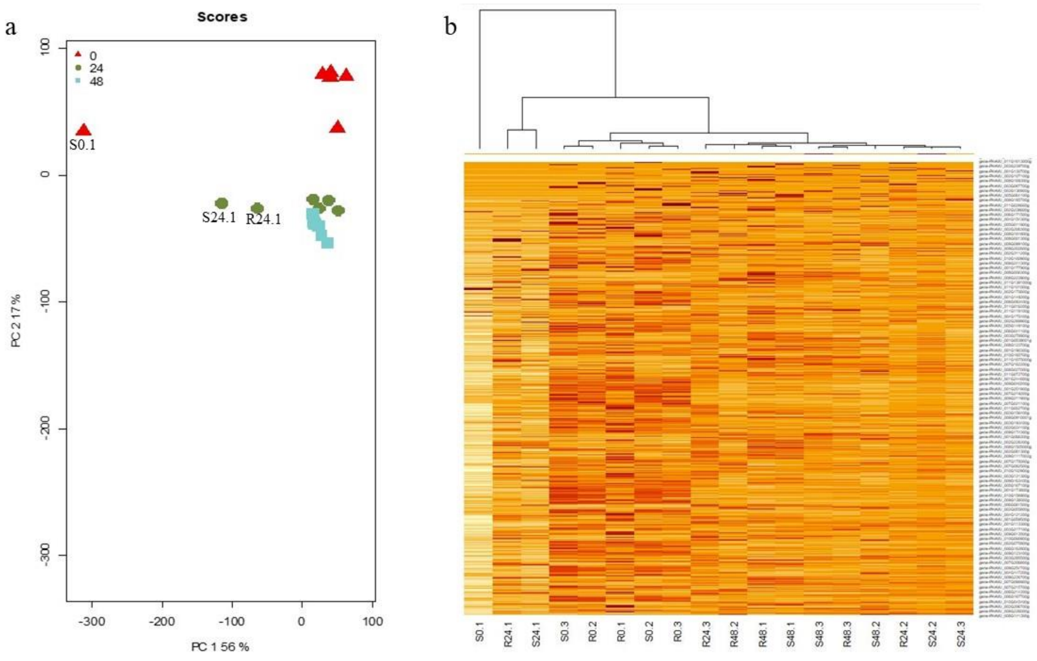

Supplementary Figure 2. RNA seq quality exploration. a) Scatterplots of the two principal components over FPKM normalized data contains all the samples. b) Heatmap and hierarchical clustering analysis (HCA) of FPKM values contains all samples.

3A

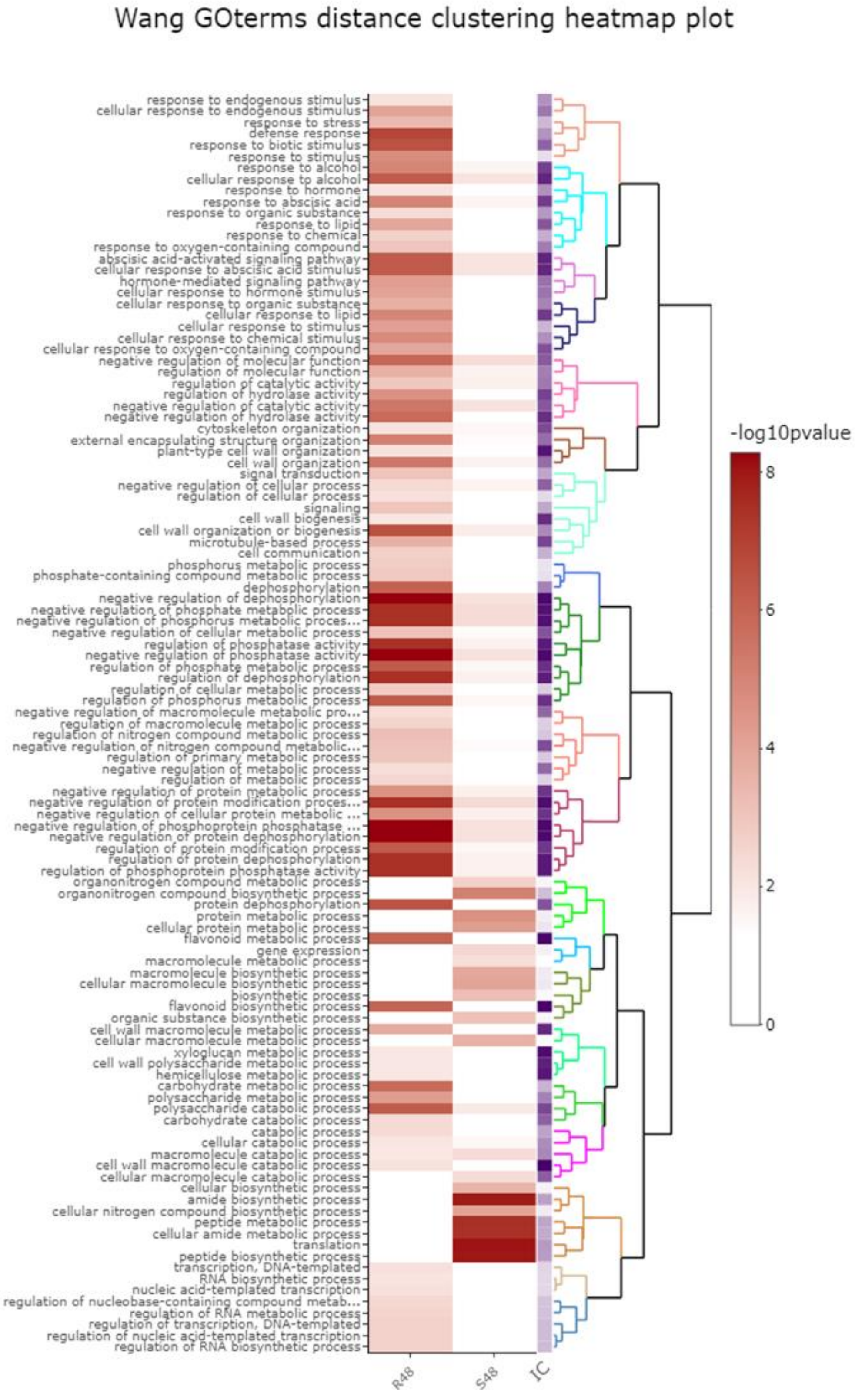
Wang GOterms distance clustering heatmap plot
response to endogenous stimulus
cellular response to endogenous stimulus
response to stress
defense response
response to biotic stimulus
response to stimulus
response to alcohol
cellular response to alcohol
response to hormone
response to abscisic acid
response to organic substance
response to lipid
response to chemical
response to oxygen-containing compound
abscisic acid-activated signaling pathway
cellular response to abscisic acid stimulus
hormone-mediated signaling pathway
cellular response to hormone stimulus
cellular response to organic substance
cellular response to lipid
cellular response to stimulus
cellular response to chemical stimulus
cellular response to oxygen-containing compound
negative regulation of molecular function
regulation of molecular function
regulation of catalytic activity
regulation of hydrolase activity
negative regulation of catalytic activity
negative regulation of hydrolase activity
cytoskeleton organization
external encapsulating structure organization
plant-type cell wall organization
cell wall organization
signal transduction
negative regulation of cellular process
regulation of cellular process
signaling
cell wall biogenesis
cell wall organization or biogenesis
microtubule-based process
cell communication
phosphorus metabolic process
phosphate-containing compound metabolic process
dephosphorylation
negative regulation of dephosphorylation
negative regulation of phosphate metabolic process
negative regulation of phosphorus metabolic proces...
negative regulation of cellular metabolic process
regulation of phosphatase activity
negative regulation of phosphatase activity
regulation of phosphate metabolic process
regulation of dephosphorylation
regulation of cellular metabolic process
regulation of phosphorus metabolic process
negative regulation of macromolecule metabolic pro...
regulation of macromolecule metabolic process
regulation of nitrogen compound metabolic process
negative regulation of nitrogen compound metabolic...
regulation of primary metabolic process
negative regulation of metabolic process
regulation of metabolic process
negative regulation of protein metabolic process
negative regulation of protein modification proces...
negative regulation of cellular protein metabolic ...
negative regulation of phosphoprotein phosphatase ...
negative regulation of protein dephosphorylation
regulation of protein modification process
regulation of protein dephosphorylation
regulation of phosphoprotein phosphatase activity
organonitrogen compound metabolic process
organonitrogen compound biosynthetic process
protein dephosphorylation
protein metabolic process
cellular protein metabolic process
flavonoid metabolic process
gene expression
macromolecule metabolic process
macromolecule biosynthetic process
cellular macromolecule biosynthetic process
biosynthetic process
flavonoid biosynthetic process
organic substance biosynthetic process
cell wall macromolecule metabolic process
cellular macromolecule metabolic process
xyloglucan metabolic process
cell wall polysaccharide metabolic process
hemicellulose metabolic process
carbohydrate metabolic process
polysaccharide metabolic process
polysaccharide catabolic process
carbohydrate catabolic process
catabolic process
cellular catabolic process
macromolecule catabolic process
cell wall macromolecule catabolic process
cellular macromolecule catabolic process
cellular biosynthetic process
amide biosynthetic process
cellular nitrogen compound biosynthetic process
peptide metabolic process
cellular amide metabolic process
translation
peptide biosynthetic process
transcription, DNA-templated
RNA biosynthetic process
nucleic acid-templated transcription
regulation of nucleobase-containing compound metab...
regulation of RNA metabolic process
regulation of transcription, DNA-templated
regulation of nucleic acid-templated transcription
regulation of RNA biosynthetic process
R48
S48
IC
-log10pvalue
8
6
4
2
0

A

3B

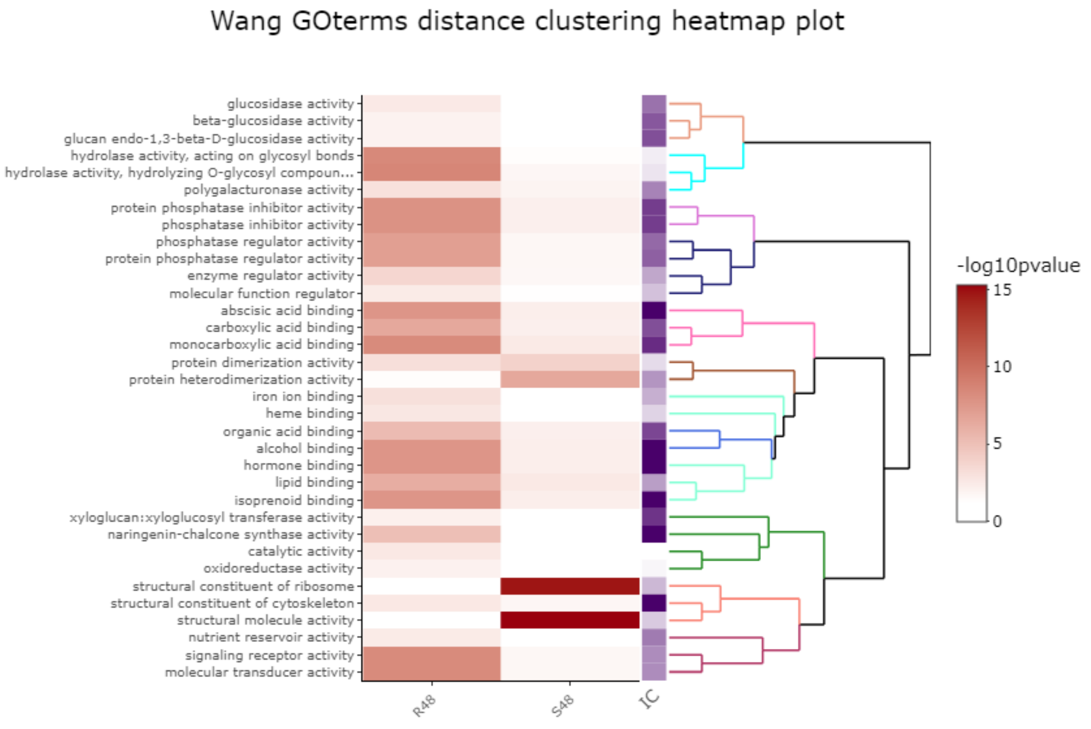

Wang GOterms distance clustering heatmap plot
glucosidase activity
beta-glucosidase activity
glucan endo-1,3-beta-D-glucosidase activity
hydrolase activity, acting on glycosyl bonds
hydrolase activity, hydrolyzing O-glycosyl compoun...
polygalacturonase activity
protein phosphatase inhibitor activity
phosphatase inhibitor activity
phosphatase regulator activity
protein phosphatase regulator activity
enzyme regulator activity
molecular function regulator
abscisic acid binding
carboxylic acid binding
monocarboxylic acid binding
protein dimerization activity
protein heterodimerization activity
iron ion binding
heme binding
organic acid binding
alcohol binding
hormone binding
lipid binding
isoprenoid binding
xyloglucan:xyloglucosyl transferase activity
naringenin-chalcone synthase activity
catalytic activity
oxidoreductase activity
structural constituent of ribosome
structural constituent of cytoskeleton
structural molecule activity
nutrient reservoir activity
signaling receptor activity
molecular transducer activity
R48
S48
IC
-log10pvalue
15
10
5
0

3C

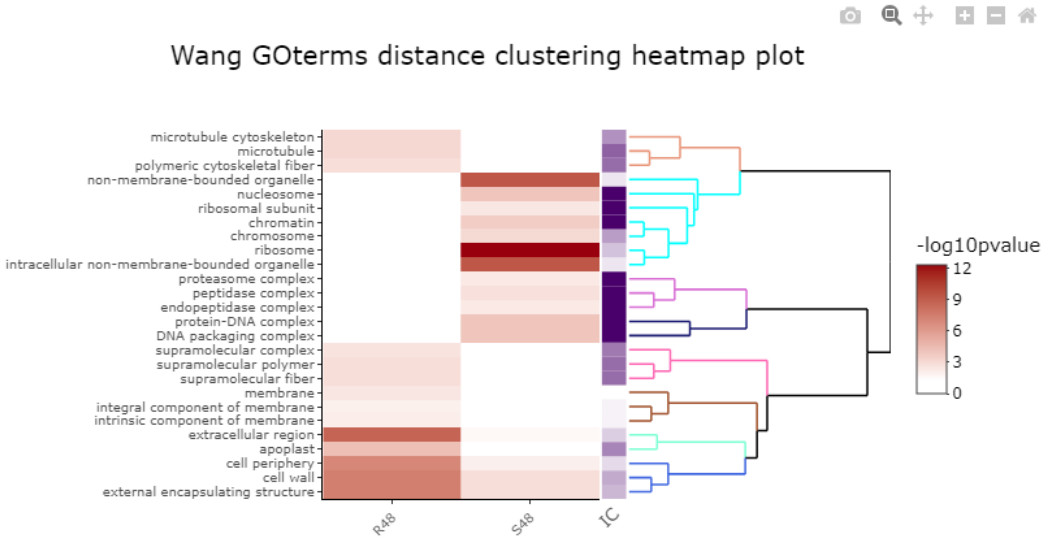

Wang GOterms distance clustering heatmap plot
microtubule cytoskeleton
microtubule
polymeric cytoskeletal fiber
non-membrane-bounded organelle
nucleosome
ribosomal subunit
chromatin
chromosome
ribosome
intracellular non-membrane-bounded organelle
proteasome complex
peptidase complex
endopeptidase complex
protein-DNA complex
DNA packaging complex
supramolecular complex
supramolecular polymer
supramolecular fiber
membrane
integral component of membrane
intrinsic component of membrane
extracellular region
apoplast
cell periphery
cell wall
external encapsulating structure
R48
S48
IC
-log10pvalue
12
9
6
3
0

3D

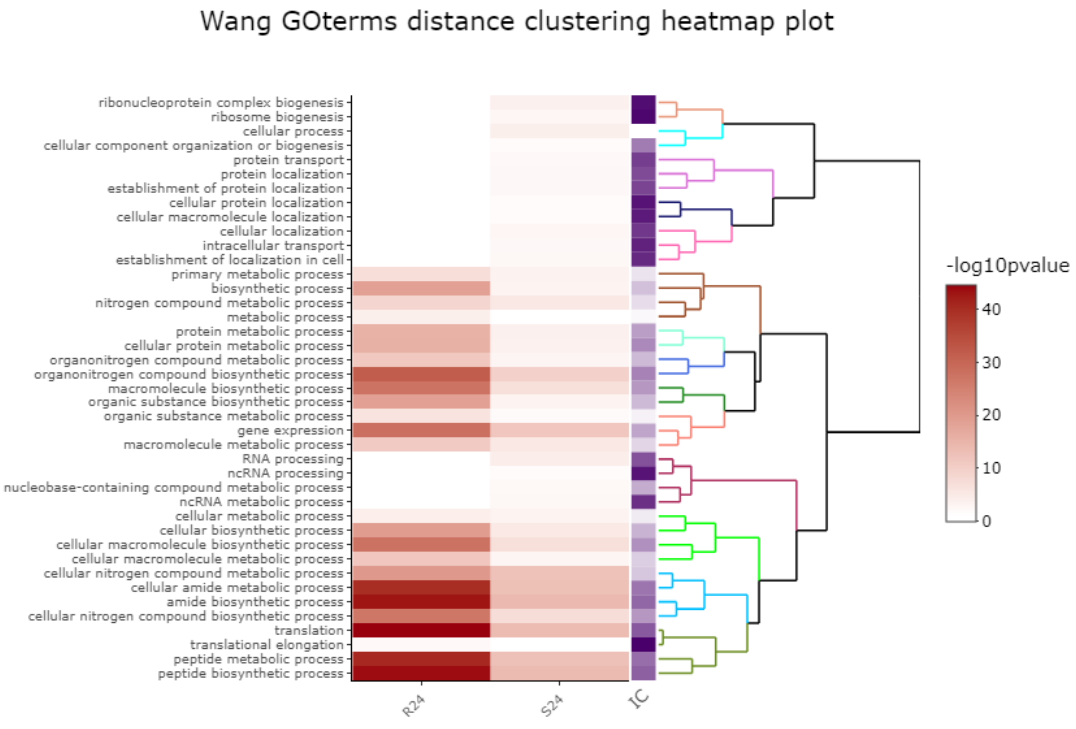
Wang GOterms distance clustering heatmap plot
ribonucleoprotein complex biogenesis
ribosome biogenesis
cellular process
cellular component organization or biogenesis
protein transport
protein localization
establishment of protein localization
cellular protein localization
cellular macromolecule localization
cellular localization
intracellular transport
establishment of localization in cell
primary metabolic process
biosynthetic process
nitrogen compound metabolic process
metabolic process
protein metabolic process
cellular protein metabolic process
organonitrogen compound metabolic process
organonitrogen compound biosynthetic process
macromolecule biosynthetic process
organic substance biosynthetic process
organic substance metabolic process
gene expression
macromolecule metabolic process
RNA processing
ncRNA processing
nucleobase-containing compound metabolic process
ncRNA metabolic process
cellular metabolic process
cellular biosynthetic process
cellular macromolecule biosynthetic process
cellular macromolecule metabolic process
cellular nitrogen compound metabolic process
cellular amide metabolic process
amide biosynthetic process
cellular nitrogen compound biosynthetic process
translation
translational elongation
peptide metabolic process
peptide biosynthetic process
R24
S24
IC
-log10pvalue
40
30
20
10
0

3E

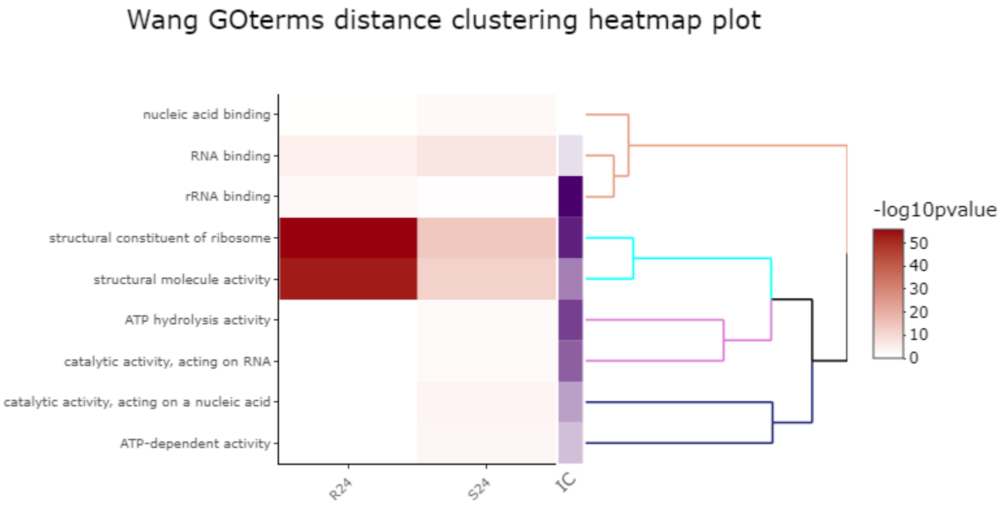
Wang GOterms distance clustering heatmap plot
nucleic acid binding
RNA binding
rRNA binding
structural constituent of ribosome
structural molecule activity
ATP hydrolysis activity
catalytic activity, acting on RNA
catalytic activity, acting on a nucleic acid
ATP-dependent activity
R24
S24
IC
-log10pvalue
50
40
30
20
10
0

3F

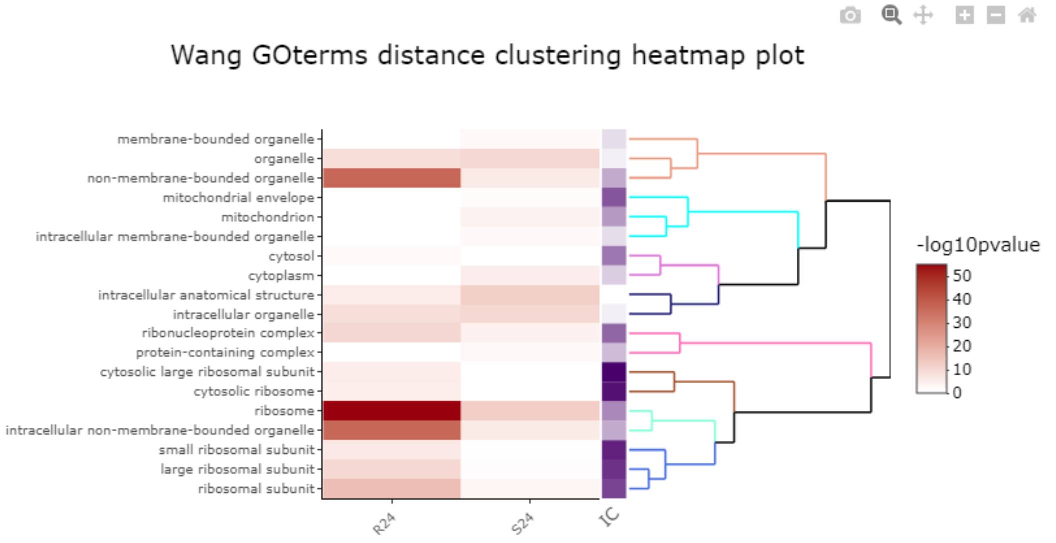

Supplementary Figure 3 (go to file). Visualization of ViSEAGO´s functional analysis from 1899 DEGs with assigned GO for the categories 'Biological process (BP)', 'Molecular Function (MF)', and 'Cellular Components (CC)'. Clustering heatmap plot that combines a dendrogram based on Wang's semantic similarity distance and ward.D2 aggregation criterion, a heatmap of -log10(p-value) from functional enrichment tests and the information content (IC). **Supplementary Figure 3a** Functional enrichment terms for BP at 48 hpi in the two genotypes (DEGs from comparations R0-R48 and S0-S48, respectively); **Supplementary Figure 3b** Functional enrichment term for MF at 48 hpi; **Supplementary Figure 3c** Functional enrichment terms for CC at 48 hpi; **Supplementary Figure 3**d Functional enrichment term for BP at 24 hpi; **Supplementary Figure 3**e Functional enrichment term for MF at 24 hpi; **Supplementary Figure 3f** Functional enrichment term for CC at 24 hpi.

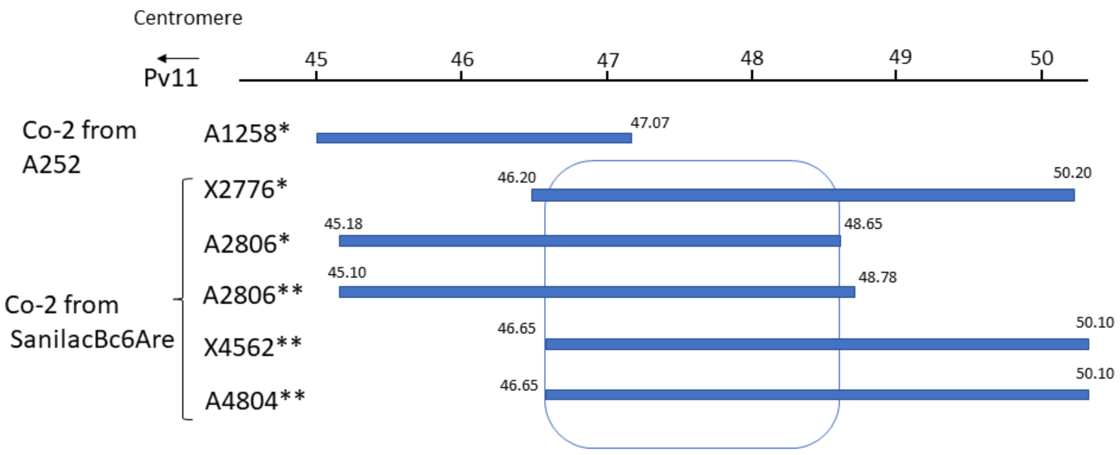

Supplementary Figure *4*. The end of bean chromosome Pv11 with the introgression regions (SNPs with genotypes SanilacBc6 Are) in the NILs A1258, X2776, A2806, and A4804. * GBS results of Murube et al (2017) ** GBS results of the present study

5A

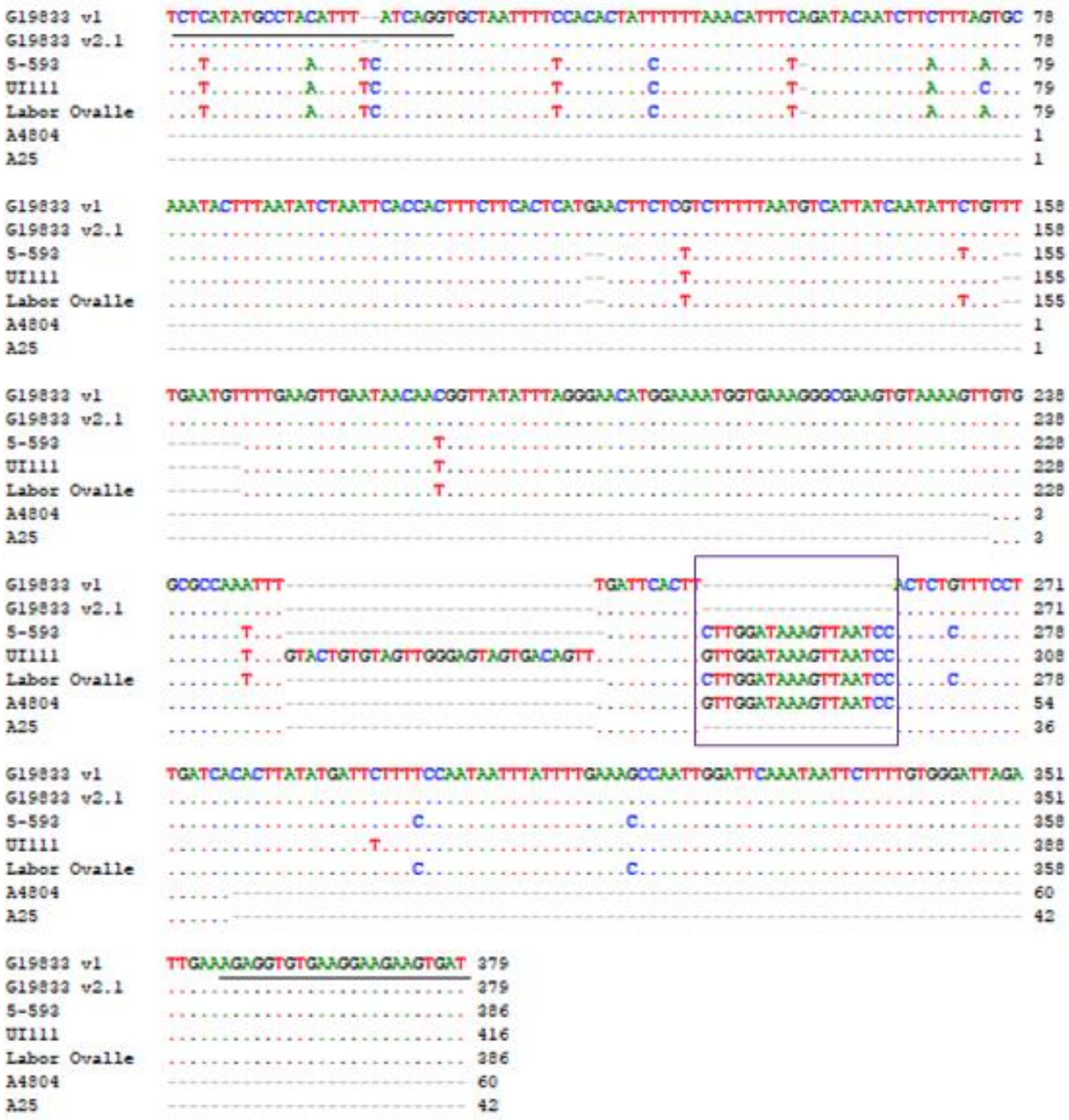

5B

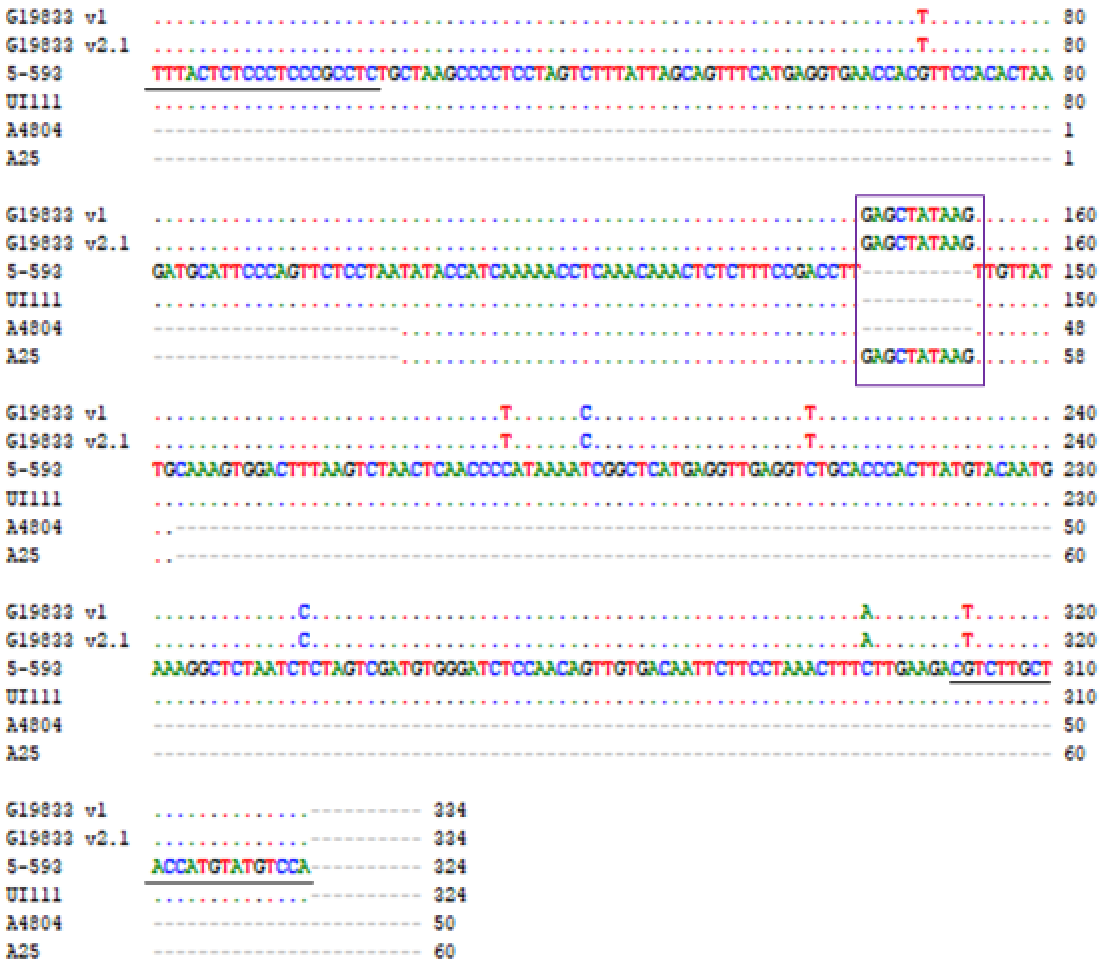
G19833 v1
G19833 v2.1
5-593
UI111
A4804
A25
TTTACTCTCCCTCCCGCCTCTGCTAAGCCCCTCCTAGTCTTTATTAGCAGTTTCATGAGGTGAACCACGTTCCACACTAA
T
80
1
GATGCATTCCCAGTTCTCCTAATATACCATCAAAAACCTCAAACAAACTCTCTTTCCGACCTT
TTGTTAT
GAGCTATAAG
160
150
48
58
TGCAAAGTGGACTTTAAGTCTAACTCAACCCCATAAAATCGGCTCATGAGGTTGAGGTCTGCACCCACTTATGTACAATG
T
C
240
230
50
60
AAAGGCTCTAATCTCTAGTCGATGTGGGATCTCCAACAGTTGTGACAATTCTTCCTAAACTTTCTTGAAGACGTCTTGCT
A
320
310
ACCATGTATGTCCA
324

5C

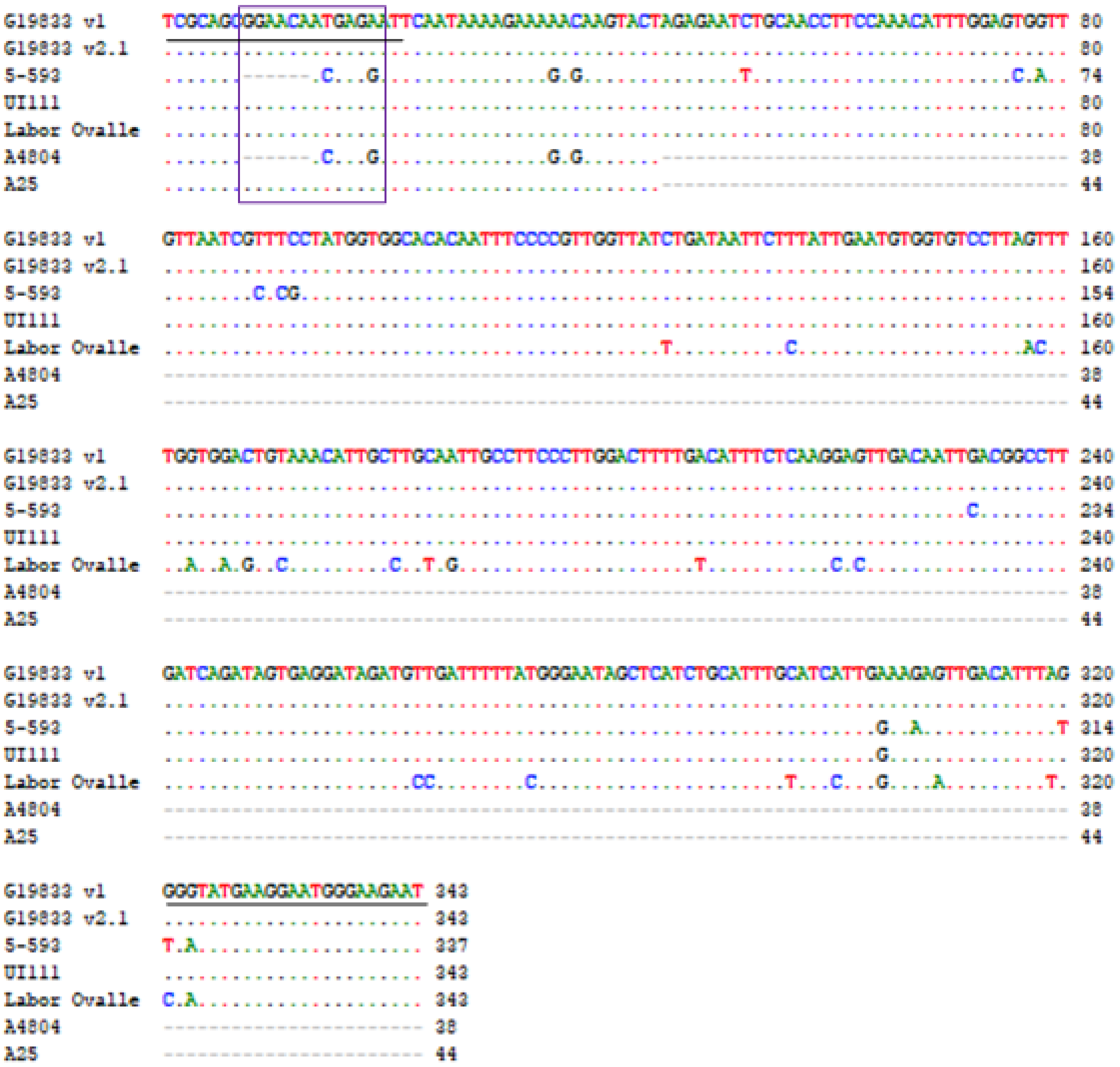

5D

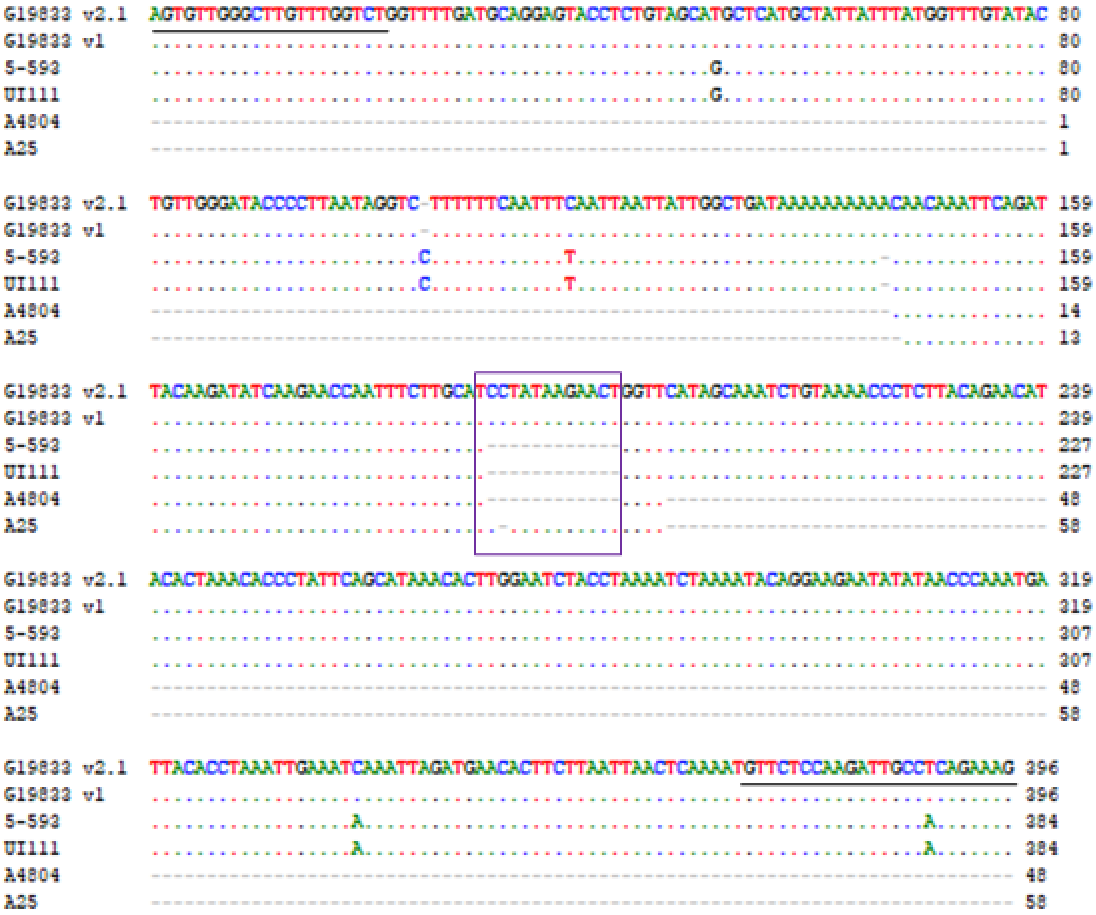

Supplementary Figure 5. Results of the alignments of four read obtained in the genotypes A25 (susceptible) and A4804 (resistant) with the bean genomes G19833v1, G19833v2.1, 5-592, UI111 and Labor Ovalle (https://phytozome-next.jgi.doe.gov/). Used polymorphisms to develop specific molecular markers are indicated in the box. Sequences and positions of the forwards and reverses primers are shown as underlines /A) M1 - Co2_46.961.315. B) M2 - Co2_46.984.860. C) M3 - Co2_46.989.310. D) M4 - Co2_47.017.090.

Supplementary Table 1. Quality of RNA sequencing data. Sample nomenclature: S, susceptible genotype A25; R, resistant genotype A4804; 0, 24, 48, hpi; 1, 2, 3, repetitions (different resistance tests).

Sample	RIN	Raw reads	Trimmed Reads	Read mapping ratio (%)	GC(%)	Q20(%)
R0.1	6.5	59,678,300	58,983,686	89.2	47.34	98.4
R0.2	4.8	47,954,978	47,643,426	91.6	47.92	98.68
R0.3	6	55,538,496	55,053,958	88.2	48.48	98.54
R24.1	5.7	59,841,376	58,867,234	82.1	50.47	97.84
R24.2	6.6	46,890,776	46,532,174	95.9	45.68	98.6
R24.3	7.1	51,323,166	50,949,782	94.4	44.16	98.38
R48.1	7.2	49,273,624	48,854,130	93.8	45.6	98.01
R48.2	6.3	41,971,556	41,630,354	93.5	47.23	98.58
R48.3	6.7	42,635,410	42,380,462	94.9	47.05	98.73
S0.1	4.3	59,679,422	58,853,356	79.7	54.73	98.04
S0.2	5.2	44,027,302	43,738,272	89.3	48.18	98.71
S0.3	6	40,169,052	39,796,838	91.4	47.2	98.4
S24.1	5.2	59,459,160	58,554,648	81.5	52.18	98.04
S24.2	6.2	40,205,802	39,895,492	95.6	46.08	98.54
S24.3	6.1	44,949,436	44,601,240	95	46.64	98.6
S48.1	7.2	47,361,130	46,902,620	91.9	46.18	97.92
S48.2	6.7	49,826,690	49,459,354	95.9	47.24	98.58
S48.3	6.8	44,398,226	44,059,632	95.4	46.94	98.63

Supplementary Table 2 (see Excel file). List of differential genes expressed observed in the seven in the nine comparisons made (R24-R0, R48-R0, S24-S0, S48-S0, R0-S0, R24-S24 and R48-S48) using the package NOISeq. FPKM (Fragments Per Kilobase of transcript per Million Mapped reads); M (which is the log2-ratio of the two conditions) and D (the value of the difference between conditions). GO (Gene Ontology) assigned to each gene are also indicated.

Supplementary Tables 3 (see Excel file) List of GO terms significantly enriched at 48 hpi revealed by the package ViSEAGO. a) List of GO terms significantly enriched for Biological Functions (**see Supplementary Figure** 3a), b) List of GO terms significantly enriched for Molecular Functions (see **Supplementary Figure** 3b), c) List of GO terms significantly enriched for Cellular Component (see **Supplementary Figure** 3c)

Supplementary Table 4 (see Excel file) List of GO terms significantly enriched at 24 hpi revealed by the package ViSEAGO. a) List of GO terms significantly enriched for Biological Functions (see **Supplementary Figure** 3d), b) List of GO terms significantly enriched for Molecular Functions (see **Supplementary Figure** 3e), c) List of GO terms significantly enriched for Cellular Component (see **Supplementary Figure** 4f)

Supplementary Table 5. Characteristics of the markers developed from the raw read sequences revealed by transcriptome analysis. Forward and reverse primer sequence, primer annealing temperature (AT) that produced the PCR products. Markers were named with Co2 following by the position in the chromosome 11 of the polymorphism to be amplified in *Phaseolus vulgaris* v1.

Polymorphism amplified	Marker name		Sequence (5' -> 3')	AT (ºC)	Expected size (bp)
1	**Co2_46.961.315**	Forward	M1F TCTCATATGCCTACATTTATCAGGT	60.9	379
		Reverse	M1R ATCACTTCTTCCTTCACACCTCT	61.1	
2	**Co2_46.984.860**	Forward	M2F TTTACTCTCCCTCCCGCCTC	62.5	334
		Reverse	M2R TGGACATACATGGTAGCAAGAAG	61.1	
3	**Co2_46.989.310**	Forward	M3F TCGCAGCGGAACAATGAGAAT	59.4	343
		Reverse	M3R ATTCTTCCCATTCCTTCATACCCC	63.5	
4	**Co2_47.017.090**	Forward	M4F AGTGTTGGGCTTGTTTGGTCT	59.4	396
		Reverse	M4R CTTTCTGAGGCAATCTTGGAGAAC	63.5	
